# The Balancing Act of Involving Relatives of Hospitalised Patients: A Qualitative Study of Attitudes, Expectations and Involvement Among Patients, Relatives and Healthcare Professionals

**DOI:** 10.1111/scs.70328

**Published:** 2026-10-21

**Authors:** Mona Kyndi Pedersen, Sofie Ladekarl Bøtker, Kirstine Cecilia Ladefoged Nielsen, Karin Dam Eikhof, Ann Karenina Kløve Laugaard, Mettine Thorsmark Madsen, Daniel Krag Nielsen

**Affiliations:** ^1^ Centre for Clinical Research, North Denmark Regional Hospital Hjoerring Denmark; ^2^ Department of Clinical Medicine Aalborg University Aalborg Denmark; ^3^ Department of Nursing University College of Northern Denmark Aalborg, Hjoerring and Thisted Denmark

**Keywords:** hospital care, Participatory Action Research, patient‐centred care, qualitative research, relative involvement, strategies

## Abstract

**Aims and Objectives:**

This study aims to describe the current practices for involving relatives of hospitalised patients.

**Methodological Design and Justification:**

A descriptive qualitative design using a combination of field observations and semi‐structured individual interviews with hospitalised patients, relatives and healthcare professionals was applied as part of the first phase of a multiphase Participatory Action Research project. The Consolidated Criteria for Reporting Qualitative Research (COREQ) guided the reporting.

**Ethical Issues and Approval:**

The study adhered to the Helsinki Declaration and Nordic ethical guidelines for nursing research. All participants provided informed consent.

**Outcome Measures and Findings:**

Three overarching themes emerged: different strategies and approaches for interaction between relatives and healthcare providers, various roles and positions of relatives and enablers and barriers for involvement of relatives.

**Study Limitations:**

The study is limited by a small number of interviewed patients and relatives (all spouses) which may limit the transferability of findings to other types of relatives, such as adult children, as well as to close friends and similar relations, who may have different roles and expectations.

**Conclusions:**

Involvement of relatives in hospital care is a complex balancing act, shaped by professional attitudes and the evolving expectations of patients and relatives. Our findings reveal that relatives occupy shifting roles and may require support themselves, with both organisational and personal factors influencing their involvement. Consequently, recognising the diverse roles and support needs of relatives is essential for preventing misunderstandings and for person‐ and family‐centred care.

## 1 Background

### 1.1 Introduction

Globally, systematic involvement of patients and their relatives in care and treatment is an important and fundamental aspect of quality efforts within healthcare [1]. Relatives such as spouses, adult children and siblings, as well as friends and neighbours, often act as both social support and resource persons for a hospitalised patient [2, 3, 4]. Due to their clinical condition, hospitalised patients are often unable to participate actively in the management of their own disease and illness [5]. In these situations, relatives can provide healthcare professionals (HCPs) with insight into valuable knowledge about the patients' medical history, preferences and personal values, as well as relating information that the patient may be unwilling to share [4, 6, 7, 8], which is also appreciated by the HCPs [9]. Research indicates that relatives in general want to be involved in patient care, and patients and their relatives must be considered as a unity [4]. Collaborative communication leading to shared expectations between HCPs, patients and their families reduces stress to patients and families [10], which facilitates effective care planning and in turn reduces pressure on the healthcare system [11]. Even though patients, relatives and HCPs benefit from involvement and collaboration, there has been limited attention to strategies and approaches for how to systematically and proactively involve relatives of the hospitalised patient [12].

When relatives feel they are seen by HCPs and valued for their presence, they often perceive themselves as involved [4], though unfortunately some relatives report feeling invisible and unrecognised by HCPs, and also that they are met with evasiveness from HCPs and absence of fundamental care for the hospitalised patient [5, 10]. For HCPs, these challenges may be related to unpredictability and uncertainty of the relative's needs and preferences of participation [13]. The position and role of the relative in health care are often unclear to HCPs, the patients and the relatives themselves [13]. Research indicates that first impressions matter for the subsequent relationship between HCPs and relatives, because an HCP has the opportunity to build mutual respect by acknowledging the patient and the relatives [14], which makes it essential for HCPs to have an understanding of the experiences and the perspectives of the relatives.

### 1.2 Aim

With an insight into the importance of involving the relatives of hospitalised patients, the aim of this study was to describe the current practice of involvement of relatives of hospitalised patients. The focus is on:

- What are the strategies and approaches for the involvement of relatives used by healthcare professionals?
- What are relatives' experiences of their role and positions?
- Which conditions enable or hinder the involvement of relatives in hospital care?

## 2 Methods and Design

### 2.1 Research Design

A descriptive qualitative design was used for this study. The Consolidated Criteria for Reporting Qualitative Research (COREQ) guidelines guided reporting in this study [15].

The study is the descriptive part of a multiphase qualitative Participatory Action Research study aiming to strengthen the collaboration between HCPs and relatives of hospitalised patients through employing user involvement processes [16]. A patient representative was part of the research group and participated in the entire research process (e.g., contributed to piloting the interview guide, provided feedback on strategies for recruitment of participants, and provided information materials prepared for patients and relatives), and another patient representative was part of the steering group.

### 2.2 Settings

The study was conducted at a medical and a surgical ward at a regional hospital in northern Denmark. Involvement of relatives has a political focus nationally as well as in the North Denmark Region and at the hospitals. The participating wards voluntarily signed up for this project, as prior to the project they were motivated and interested in researching involvement of relatives. The inclusion of multiple settings was intended to capture shared experiences between patient populations, clinical practices and HCPs and to describe patterns across different medical specialties rather than to compare wards systematically.

### 2.3 Data Collection and Participants

To provide a comprehensive understanding of the involvement of relatives, data were collected using a combination of field observations [17] and semi‐structured individual interviews [18] with hospitalised patients, relatives and HCPs. Data were collected in a 2‐month period from November 2021 to December 2021. The field observations and individual interviews were conducted by two authors (KDE and AKKL) who had previous experiences with qualitative methods such as interviews and observations. Neither of the interviewers had prior relationships with any of the participants. Three observations (each of them had a duration of 5 h) were conducted at each ward during both day and evening shifts (30 h of total observation). Based on an observation guide inspired by Spradley [19] the observers followed available HCPs (doctors, nurses and social and health care assistants) to observe the current practice of involvement of relatives (Box 1). During the observations, informal interviews and reflexive conversations were conducted with HCPs. Ongoing field notes were made during the observations, supplemented with the observers' own reflections [20].

BOX 1 **Key dimensions in the observation tool.**

**Table:** 

**Actors**, including who initiates contact and engages in care. **Time and Space**, focusing on when and where interactions occur. **Activities**, encompassing the specific tasks and collaborative actions performed. **Interactions**, capturing the dynamics between patients, relatives and staff. **Reflective notes** (the observer): • What appears to be the usual practice regarding involvement of relatives? • What appears to be the staff's attitudes towards involvement of relatives? • How does the collaboration between staff, patients and relatives appear to function?

One to two weeks after the observation period, individual semi‐structured interviews with patients, relatives and HCPs were conducted. Participants selected for interviews (*n* = 6) differed from the participants in the field observations. For this study, ‘relatives’ were defined as family members and other close relations, including adult children, close friends and similar significant others, as well as any individual whom the patients themselves identified as their primary support person. Patients and relatives were invited to participate in the interviews based on accessibility and their interest to contribute by the head nurses. The interviewed HCPs were affiliated with the two wards and recruited by the head nurses based on their expressed interest, informed consent and availability, reflecting a purposive though convenience sampling approach.

The interviews were constructed to encourage the participants to reflect on their experiences with involvement of relatives or being involved as a relative. Three semi‐structured interview guides focused on either patients, relatives or HCPs were used to structure the interviews. The interview guides were discussed among the interviewers and the patient representative, and subsequently some of the questions were slightly adjusted (Box 2). Patients, relatives and HCPs (*n* = 6) affiliated to the two wards participated in the interviews, which took place in an undisturbed room at the hospital. Each interview was digitally recorded and lasted from 30 to 60 min. The interviews were verbally transcribed by an external transcriber (student nurses) or a research assistant (SLB).

BOX 2 **Key topics in the interview guides for patients, relatives and healthcare professionals.**

**Table:** 

What does the involvement of relatives mean to you, and how is it manifested during a hospital stay? What is the perceived purpose of involving relatives in the care process? What are the outcomes of this involvement for patients, relatives and healthcare professionals? Could you provide a specific example? What worked well, and what could have been improved? Who was responsible for facilitating this involvement? Do you have any recommendations on how to better involve relatives in clinical practice?

The characteristics of the participants are presented in Table 1. The participating HCPs in the field observations represented nurses, doctors and social and health care assistants (*n* = 25). The interviews involved two patients and two relatives, who were not recruited as patient–relative pairs. The sample included both female and male participants, and both relatives were spouses. For further details, see Table 1.

**TABLE 1:** Characteristics of the study participants.

Characteristics	Observations	Interviews
Patients		(*n* = 2)
Age (years, mean)		72.5
Gender (number)		
Male		1
Female		1
Other		—

Relatives		(*n* = 2)
Age (years, mean)		57
Relation (number)		
Spouse		2
Gender		
Male		1
Female		1
Other		—

Healthcare professionals	(*n* = 25)^a^	(*n* = 2)
Age (years, mean)	40	47.5
Gender (number)		
Male	2	—
Female	23	2
Other	—	—
Profession (number)		
Nurses	12	1
Care assistants	4	1
Doctors	7	—
Other	2	—
Experience at the ward (years) < 1	5	—
1–10	12	2
11–20	5	—
> 20	2	—

^a^ *n* = 12 and *n* = 13 participants from the two wards.

### 2.4 Data Analysis

The research team comprised a multidisciplinary group consisting of female and male qualitative researchers, clinical nurses, educators and a patient representative. To ensure methodological rigour and to minimise bias, a collaborative approach was used throughout the study. This included collective discussions regarding the development of the research instruments, the coding process and the interpretation of themes. By integrating perspectives from both clinical practice and the patient representative's lived experience, the team employed a reflexive process to ensure the findings accurately reflected the multifaceted nature of involvement of relatives in hospital care.

Data were derived from systematic field observations and semi‐structured interviews, and written notes were, in connection with the transcripts of the interviews, equally used as data material. These two data sources were analysed using an integrative approach, where observations provided the situational context and interviews provided the lived experience of the participants [21]. The data analysis was conducted based on reflexive thematic analysis as described by Braun and Clarke. Thematic analysis is described as a theoretically flexible stepwise approach which is suitable for research questions regarding people's views, perceptions and experiences [22, 23]. First, the process involved iterative stages of familiarisation with the data and line‐by‐line coding [23]. Four authors (SLB, KDE, AKKL and KCLN) individually coded the data material based on field observations and interviews, and afterwards a joint coding was undertaken and continuously discussed and reviewed in collaboration with the senior researchers (MKP and DKN). Second, SLB, KDE, AKKL and KCLN mapped the codes into three initial themes and sub‐themes. These themes and sub‐themes were revised together with the patient representatives by reviewing data extracts and data sets to ensure that the themes were qualified. Table 2 illustrates an example of the coding and theme developing process. Finally, the identified themes were discussed with the research group including the patient representative.

**TABLE 2:** Example of the analysis process from meaning units to the theme: Enablers and barriers for involvement of relatives.

Data extracts	Codes	Sub‐themes	Themes
The nurse is asking questions, and her husband [relative] is explaining how to use the machine [medical device]. They talk about when it should be used. The nurse asks if it should also be used when the patient takes a nap during the day. The husband says that could be a good idea. They chat back and forth and both agree that using the mask during the midday nap would be helpful. The man shows how to put it on the patient, fumbling a bit with the straps that keep it in place. The nurse steps in, and they work together to get it done properly. (Observation, evening shift) ‘Yeah, because we really like to involve them [the relatives]. And it's just great when we can make it work—when the patient wants to be part of the treatment and the relatives want to be here too. And we can kind of get that collaboration going.’ (Healthcare professional) ‘When relatives are present, they bring a different view of the patient that we don't know, and they can give us a lot of information we might not have been aware of—and we need to act on that too. So, I guess that's… why it [involving relatives] can sometimes feel like a disruption. But I really can't see how you could provide optimal care without involving the relatives, if they're there and willing. That would be optimal care.’ (Healthcare professional)	Relatives as a resource	Healthcare providers meet the relatives with an open approach and see the value in relatives as a resource	Enablers and barriers for the involvement of relatives
‘He wouldn't cooperate with me until the nurse took my side and said, ‘You have to listen’. And then it was two against one.’ (Relative) ‘The relative attended the ward round seeking information about the upcoming care. The interaction between the physician, the patient and the relative appeared rushed, as the physician provided brief information before quickly exiting the room.’ (Observation)	Relatives' needs are dismissed	Healthcare providers have lack of focus on the involvement of relatives	

### 2.5 Ethical Issues

The study was conducted according to ethical principles from the Helsinki Declaration [24] and the Ethical Guidelines for Nursing Research in the Nordic Countries [25]. The participants received both oral and written information about the study and were promised confidentiality and anonymity before signing a consent form and before the interviews or the observations were made. All participants were notified that they could withdraw their consent at any time without any reason or consequence. None of the authors were employed in the participating wards. None of the participants withdrew from the study. Data were handled confidentially and anonymously in the presentation of findings.

## 3 Findings

The following presentation of findings describes the current practice for involvement of relatives at the hospital wards under study and provides insight into the experiences of relatives, patients and HCPs regarding involvement of relatives. The findings encompass the following themes: *Different strategies and approaches for interaction between relatives and healthcare providers, Various roles and positions of relatives* and *Enablers and barriers for involvement of relatives*. The themes are presented in Table 3. These themes describe the participants' actions, understanding and considerations about involvement of relatives. In the presentation of themes, quotations from the interviews provide empirical support for the themes and capture the nuances of the participants' lived experiences.

**TABLE 3:** Overview of themes and sub‐themes.

Theme	Sub‐themes
Different strategies and approaches for interaction between relatives and healthcare providers	Established methods for involving relatives
	Everyday conversation with patients and relatives
	Relatives are seeking involvement
Various roles and positions of relatives	Relatives see themselves as helpers to the patient
	Relatives see themselves as helpers to the healthcare providers
	Relatives are also affected by the hospitalisation
	Not everyone wants to be involved
Enablers and barriers for involvement of relatives	Healthcare providers have lack of focus on the involvement of relatives
	Healthcare providers' perspectives on involvement of relatives
	Organisation of the department
	Healthcare providers meet the relatives with an open approach and see the value in relatives as a resource

### 3.1 Different Strategies and Approaches for Interaction Between Relatives and Healthcare Providers

HCPs used different approaches to involve relatives and various circumstances either complicated or promoted the level of involvement. The HCPs used formal, systematic approaches where relatives participated in different events such as rounds, goalsetting, discharge conversations and active participation in daily care. The HCPs tried to involve the relatives in situations whenever possible:

I'm not really involved in ward rounds and that kind of stuff, but I do the day‐to‐day care. And that's where I try to involve the relatives as well, you know, to make them feel like they're part of the hospital stay too, together with the patient. That they're seen and heard as well. That's important. (Healthcare professional)

More informal, casual ways for involving relatives were also used, which were appreciated by patients, relatives and HCPs. Small talk and everyday conversation with the relatives made them feel involved:

Small talk is incredibly important. It really matters that she [the nurse] asks about things like, do you have kids – what's happening with them? Oh, I've got kids too… It's really important that everything doesn't just revolve around the illness. Because otherwise you can very easily end up digging yourself deeper and deeper down. (Relative)

Relatives were also actively seeking involvement by reaching out to the HCPs through both telephone and physical contact. Some relatives made efforts to become actively involved, which was welcomed by the HCP: ‘*If you actually make an effort to be part of this, I think they're really good at letting you be involved, you know*’ (Relative). Still, occasionally relatives had to make a persistent effort to obtain the involvement that they wanted. Not all HCPs wanted to cooperate, and some needed to be forced to engage with the relatives:

The last doctor who came in to talk, he just made a quick comment, asking if I was a healthcare professional… But, I mean, you could kind of push him into cooperating, I just had to [pause] be persistent enough, and then we got a collaboration going. Whereas the other two doctors were just outright dismissive. (Relative)

### 3.2 Various Roles and Positions of Relatives

Relatives experienced shifting roles and needs for involvement in the care and treatment process during hospitalisation. The relatives describe themselves as helpers for both the patient and the HCPs. Their role as helper for the HCPs included sharing knowledge about the patient before, during and after admission and executing practical tasks to ease the staff. A patient described how his wife could help the staff to obtain insight into the period before admission:

She [my wife] could tell [the healthcare professionals] how things had been at home and things like that… She told [them] how I had been during the night… it just came on suddenly at night, where I had diarrhoea and was throwing up… and I was freezing and shaking all over my body, completely. (Patient)

The relatives wanted to ease the job of the HCPs, so they could have time for a break:

It can take a load off the staff. They don't have to worry about not being able to sit down and eat, because they don't need to run to the patient to check on them all the time [when relatives are around]. (Relative)

When it came to the role as helper for the patient, the relatives described themselves as being an extra pair of eyes and ears in situations where the patient needed it. The presence of the relatives increased the comfort and wellbeing of the patient:

It gives the patient more peace of mind to have someone they know here… they don't have to be so on edge when the staff are around, because there's someone there to help – an extra set of eyes and ears. They can focus on themselves, instead of just having to listen and remember everything that's being said. (Relative)

A nurse described how she considered the presence of the relatives as an advantage, because they could often assist the patient by remembering more information:

…when you're a patient and you get a lot of information, for example during ward rounds, you don't always catch everything yourself. And I… think it can be an advantage if a relative is there to hear it too. Many times, they end up getting a bit more information than if it was just the patient alone. (Healthcare professional)

In addition, the relatives also expressed their own feelings that they were emotionally affected by the hospitalisation. The relatives appreciated and felt supported by the HCPs, and they were confident showing their feelings to them. Sometimes the HCPs offered the relative a personal space when they were concerned or felt exhausted:

If it's the kind of thing where you need to step outside and just fall apart for five minutes… it's… so nice that someone comes over and says, ‘Look, I'll take over for a bit, this is completely normal. Don't worry, you'll be fine. We'll take care of him.’ Just like the nurse says: ‘I'll go in and check on him…’ because they know I'm really worried. (Relative)

A patient described how the HCPs offered him emotional support after what he had been through, but instead he thought of his wife, because she was the one who had been there and seen it all:

The doctor did ask me if I should get any psychological support, but I don't think so… you never really know, of course. But then I thought afterwards, it was probably my wife [the relative] who needed support [laughs], she's the one who saw what happened when I was taken out on an emergency call. (Patient)

Not all participants shared the same excitement about being involved and expressed that they did not feel the need to be involved in any way:

I'm probably from the generation where relatives weren't really involved. Except when it was necessary… I don't expect anyone to call me and say, ‘Now we're doing this, and now we're doing that.’ I've never had those expectations [laughs]. (Relative)

Similarly, a patient described that she did not want the HCPs to involve her relatives.

### 3.3 Enablers and Barriers for Involvement of Relatives

The position and level of involvement of relatives in the wards was influenced by different enablers and barriers, and relatives as well as HCPs described factors that affected the involvement of relatives. When the relatives were met with kindness, acknowledgment and open‐mindedness by the HCPs, they felt more involved. When the relatives were recognised as a valued resource by the HCPs or patients, the relatives felt involved. One relative expressed that she felt involved when she was given information, supported and included in the care of the patient: ‘*Involving relatives is largely about information… and then it's about support. And then it's about… inclusion*’ (Relative).

Some HCPs considered the relatives as a resource. Communication and collaboration were described as something mutual because the HCPs also needed to obtain knowledge and support from the relatives:

So, we use them as a resource, because we also call them. We call to check, you know, if they can come up and talk, or we ask if someone can pick up the patient – or if they have the medication at home… So, we really do call the relatives a lot. (Healthcare professional)

A nurse described how as colleagues they agreed that it is important to involve the relatives and that they actively tried to include them, which sometimes required an extra effort:

Well, I… think that we're all pretty much in agreement that we really want to involve the relatives. I think we all make an extra effort and do a lot of work to involve them. (Healthcare professional)

Barriers to involvement of relatives includes both individual and organisational barriers. Barriers encompassed individual HCPs' hesitant attitudes towards involvement of relatives. Organisational barriers at the wards also negatively influenced the approach of how to involve the relatives. Relatives expressed that they felt disregarded and not involved and that HCPs did not pay any attention to them. One relative described that she did not receive any information regarding her hospitalised husband because the HCP had already explained the procedure to the patient and did not wish to repeat this information twice:

…the first doctor who came in to look at [patient's name] – the one who just walked out again – I asked him, ‘Excuse me, what exactly did you say?’ And he said, ‘I told the patient everything,’ and then he walked out… He may have said it to the patient, but when the patient is in a lot of pain, they don't really take much in… (Relative)

Relatives described difficulties with communication and interaction with the HCPs. The relatives found it challenging to confront the HCPs when they felt neglected and had to team up with another HCP to become involved: ‘*He wouldn't cooperate with me until the nurse took my side and said, ‘You have to listen.’ And then it was two against one* *’* (Relative).

Another barrier was the attitudes regarding involvement of relatives among the HCPs, which influenced their approach, appearance and actions in relation to involving relatives negatively. An HCP described that as colleagues they do not necessarily share the same strategy and that it depends on the individual and their surplus of energy for how much the HCPs involve relatives:

…I get told off a lot by the others [colleagues], that I spend too much time on the relatives. Because, I can't help thinking, what if it were my grandma who was admitted… But again, it really depends on the individual – no matter whether you're an assistant or a nurse… some people have more emotional capacity for the relatives than others do. (Healthcare professional)

During the observations and interviews some HCPs perceived interactions with the relatives as disturbing, for example when relatives made contact with the ward during evening shifts to obtain information and updates about the patient: ‘*So you* [the interviewer] *were here the other evening, and I don't know if you noticed, but that's usually when they call in and disturb things’* (Healthcare professional).

Time–pressure also played a decisive role for the level of involvement. HCPs described how they were under time pressure and thereby did not have time to talk with the patients and their relatives:

…sometimes we're so busy that the time we have in the room with the patient and the relatives is really rushed, where you're kind of standing with one foot outside the door while trying to give information and trying to make that, what do you call it, connection with the relatives. So, it's a bit tricky, sometimes you have to be quick to make that contact, to get that trust from them or with them… (Healthcare professional)

## 4 Discussion

This paper contributes to knowledge of current strategies and approaches of involvement of relatives as well as the relatives of hospitalised patients' perception of their positions and roles. Based on field observations at the wards and interviews with relatives, patients and HCPs, we found that different formal as well as informal strategies and approaches were used to involve relatives during the hospitalisation of the patient. Our findings also illustrate different needs and attitudes to involvement among both relatives and patients. Likewise, relatives had shifting roles and positions in the care process and they themselves may also require being cared for by the HCPs. Our findings show that various factors and circumstances, both organisational and personal, either complicated or enabled involvement of relatives.

This study shows that the participating wards to some extent systematically involved patients and their families in, for example, planning meetings for goal setting and for setting realistic timeframes for the hospital stay. Likewise, more informal and spontaneous ways of interacting, such as small talk and everyday conversation, were described and highly appreciated by HCPs, patients and relatives. Research indicates that nurses' attitudes regarding the importance of family support in nursing care vary [26], which affects the involvement and in turn affects patient outcomes [27]. Personal factors including years since graduation, higher level of education and personal experiences such as having experienced illness within one's own family seem to affect the HCPs' attitudes towards involvement of relatives in a positive way [26]. Personal experience from being a relative themselves also affects the HCPs' ability to empathise with the relatives [28]. In addition, an overall explicitly proactive approach to involvement of relatives at the ward affects individual nurses' attitudes to involvement of relatives and also the role and position that relatives are given [26, 27]. Furthermore, relative involvement depends not only on individual HCPs' attitudes but also on a shared understanding of professional roles and responsibilities. Woldring et al. [29] found that overlapping and unclear role expectations among nurses and physicians may lead to inconsistent involvement and support of relatives. Together, these findings suggest that effective involvement of relatives requires a shared interprofessional understanding of the roles and responsibilities of relatives in everyday clinical practice.

In the current study, relatives perceived that it was their role to be helpful to the staff as well as to the patient. Some relatives were frustrated when they experienced difficulties in relation to collaboration with the HCPs. Sivertsen et al. [10] found that relatives of older medical patients became frustrated when they were not informed or involved in care planning or decision making for the patient. From the perspective of HCPs, there are clear benefits of involvement of relatives, as they provide valuable information about the patient while additionally helping to calm the patient [30], which is also found in the current study. However, it is also illustrated that the relatives themselves can be in a situation where they need help from the HCPs. Likewise, Guldager and colleagues describe that nurses perceive relatives both as a resource as well as resource demanding [28]. HCPs are especially challenged when dealing with relatives who express strong emotions such as distress, agitation, or even rudeness [30].

Further, in the transition between hospitals and municipal hospitals, as well as homecare, nurses experience the need for involvement of relatives due to the gap between healthcare sectors [31]. After discharge the relatives feel responsible to provide continuity in the care trajectory and they consider themselves the ‘glue’ between hospital and home that ensures that the patient's health and everyday life do not fall apart [32], which makes the findings from the current study relevant to other parts of the healthcare system. As more patients live with multimorbidity and receive care across multiple settings, relatives increasingly help coordinate care by transferring information between sectors and supporting continuity. Although they are an important resource, relying on them to ensure coherent care trajectories may also place a considerable burden on them. Our findings show that relatives' roles evolve throughout the care process and are shaped by organisational and personal factors. Recognising relatives as both contributors to care and individuals with their own support needs is therefore essential.

The roles and positions of relatives are both affected by the actions of the HCPs and the capability and actions of the relatives. Research finds that relatives take diverse positions in the interaction with the HCPs. These various positions affect to which degree the relatives interact with and relate to the HCP and the patient [33]. Some relatives describe that they must be proactive and persistent to get involved [10], which is comparable to findings in the present study. Relatives with an active and proactive profile seem to have better opportunities to interact and negotiate with HCPs than reactive, passive and hesitant relatives [34, 35]. However, this finding raises concerns about equity in involvement of relatives. When involvement depends on relatives being proactive or persistent, those less able to advocate for themselves may be excluded despite wishing to participate. HCPs should therefore not rely solely on relatives' own initiatives but actively explore their preferences, resources, and desired level of involvement. This calls for more systematic discussions about relatives' roles, rather than relying on ad hoc interactions throughout the care trajectory. In addition, another study finds that the physical, mental and social capability of the relatives appears to affect their possibility to interact and negotiate with HCPs [35]. For example, relatives' experiences about how to navigate within the healthcare system and knowledge of health‐related issues serve as enablers for involvement of relatives [35].

The involvement of relatives induces several ethical concerns for the HCPs, such as balancing the privacy of the patient with the relatives' wish for involvement [9, 36]. In the current study, some patients expressed that they did not want their relatives to be informed or involved in their care. Moreover, some relatives expressed that they did not want to become involved in the care and treatment of the patient. Therefore, another ethical concern for the HCPs is to recognise that both patients and their relatives have the right to decide whether they want to become involved or not [36]. Addressing these dilemmas requires aligning expectations among HCPs, patients and relatives while strengthening collaboration and communication. Meaningful involvement also depends on HCPs recognising relatives as legitimate partners in care rather than merely visitors or practical resources. This calls for family‐ and person‐oriented attitudes, supported by education that addresses both communication skills and the underlying values shaping HCPs' perceptions of relatives' roles. Interprofessional learning may further promote a shared understanding and a more coordinated approach to family involvement [29].

### 4.1 Methodological Strengths and Limitations

Several strengths and limitations should be considered when interpreting the findings of this study.

A primary strength lies in our methodological approach. By employing a multi‐method design that combined structured observations with in‐depth interviews, we were able to triangulate data and capture a more holistic view of involvement of relatives in the hospital environment. The observations provided essential contextual insights, while the interviews allowed for a deeper exploration of the participants' lived experiences. We included a medical and surgical ward. While differences between the two specialties in terms of professional perspectives, workflows and levels of involvement may have influenced the findings, the multi‐site context enhanced the breadth of our data. Rather than conducting a comparative study between departments, we aimed to identify universal patterns of relative involvement across different clinical settings, thereby increasing the potential transferability of our findings.

Regarding the participants, the study benefited from a diverse‐perspective approach by involving patients, relatives and HCPs. We focused on relatives who were identified by the patients themselves. This patient‐centred approach ensured that the participants represented the patients' actual social networks, rather than being limited to biological family members. As our sample of relatives consisted of spouses, we acknowledge that this may limit the transferability of the findings to other types of relatives, such as adult children or close friends, who may hold different roles and expectations.

We also acknowledge certain methodological limitations. First, the relatively small number of interviewees may raise questions regarding data saturation. However, we contend that the sample provided sufficient information power to capture rich, in‐depth perspectives [37]. Moreover, following the principles of reflexive thematic analysis, we focused on the emergence of themes through iterative interpretation rather than traditional statistical saturation [38]. Second, a potential limitation of this study is the risk for selection bias, as the participants were recruited through a method that may have favoured more proactive and engaged HCPs, patients and relatives. However, the participants who were invited to participate in the interviews showed interest in the study and wished to contribute and share their experiences with the interviewer. Consequently, the findings may not fully represent the experiences of less involved or less proactive relatives when considering the broader perspective of relative involvement.

Thirdly, the diversity of wards and clinical specialties was addressed by using a structured observation and interview guide to ensure consistency. To further mitigate potential researcher bias, two different researchers conducted the observations and interviews, and all data were analysed through a collaborative coding process where any discrepancies were discussed and resolved.

Finally, the definitions of involvement and classification into different stages and levels of involvement have long been discussed in scientific literature [39]. While we conceptualise involvement as a continuum—ranging from low to high levels of engagement [40] – we recognise that the specific roles and power dynamics between patients, relatives and HCPs are continuously negotiated [32, 35]. Thus, meaningful involvement of relatives is a dynamic process, and the success will always depend on the specific situation and the parties involved.

## 5 Conclusion

In conclusion, this study demonstrates that involving relatives in hospital care is a complex balancing act, shaped by healthcare professionals' attitudes and the varying expectations of both patients and their relatives. Our findings reveal that relatives occupy shifting roles throughout the care process and may themselves require support from clinical staff. We observed that both organisational and personal factors can facilitate or hinder meaningful involvement. Consequently, recognising the diverse roles, resources and support needs of relatives is essential to prevent misunderstanding and to foster person‐ and family‐centred care.

## 6 Implications for Clinical Practice, Education and Research

To translate these findings into practice, we recommend that clinical settings implement structured involvement strategies to explore relatives' involvement in clinical practice. In education, greater attention should be given to enhancing communication skills, professional reflection and interprofessional learning to support a coherent approach to relative involvement. The current findings from this multiphase participatory action research study will inform the subsequent phase, where strategies and targeted interventions will be developed through collaborative workshops with healthcare professionals, patients, and relatives. Finally, future research is needed to explore how these strategies and interventions can be implemented and sustained across healthcare settings, specifically examining their impact on patients, relatives and healthcare professionals.

## Author Contributions

M.K.P. and D.K.N. contributed to the conceptualisation and methodology of the study. M.K.P. drafted the manuscript in cooperation with S.L.B., M.T.M. and D.K.N. The observations and individual interviews were conducted by K.D.E. and A.K.K.L. The data material based on field observations and interviews was coded by K.C.L.N., K.D.E., S.L.B. and A.K.K.L., who were also involved in the analysis and interpretation of data and continuously discussed with the research group and reviewed in collaboration with M.K.P. and D.K.N. The first draft of the manuscript was provided by M.K.P., S.L.B., D.K.N. and M.T.M. All authors contributed to the review and critical editing and approved the final manuscript.

## Funding

This project was economically supported by a grant from Marie Pedersen and Jensine Heiberg Legat, as well as from North Denmark Region (regional fund for involvement of patients and relatives).

## Ethics Statement

The study is conducted in compliance with the General Data Protection Regulation and is a part of North Denmark Region's record of processing activities (ID 2021‐149) and University College of Northern Denmark's record of processing activities (ID 20900412). According to the Danish Health Care Act, the research project does not require any additional ethical assessment (ID: 2021‐000438).

## Consent

All participants provided informed consent.

## Conflicts of Interest

The authors declare no conflicts of interest.

## Data Availability

All relevant data are accessible in the manuscript.
